# How to slice: snapshots of Argonaute in action

**DOI:** 10.1186/1758-907X-1-3

**Published:** 2010-01-12

**Authors:** James S Parker

**Affiliations:** 1 Department of Biochemistry, University of Oxford, South Parks Road, Oxford OX1 3QU, UK

## Abstract

Argonaute is the principal protein component of the mechanisms of RNA silencing, providing anchor sites for the small guide RNA strand and the 'slicer' activity for cleavage of target mRNAs or short passenger RNA strands. Argonaute is the core constituent of the silencing effector complexes RISC (RNA-induced silencing complex) and the RITS complex (RNA-induced initiation of transcriptional gene silencing complex), interacting directly or indirectly with Dicer proteins, R2D2/Loquacious/TRBP and GW182 family proteins in the former, and Chp1 and Tas3 in the latter. In a breakthrough series of papers, Patel *et al*. provide a set of 'molecular snapshots' of the catalytic cycle of Argonaute, exploiting mismatches and mutants to capture and visualize by X-ray crystallography Argonaute from *Thermus thermophilus *with guide and target strands at various stages of the silencing process. The structural studies, coupled to structure-directed biochemical analysis, together with other thermodynamic and kinetic studies, provide insights into Argonaute with implications for the mechanisms of RNA silencing in eukaryotes.

## Introduction

Genetic and biochemical studies first implicated Argonaute (or Ago) as a key component of the mechanisms of RNA silencing in eukaryotes [1]. Argonaute proteins fall mainly into two subfamilies (Ago and Piwi), defined initially on the basis of sequence similarity [1], with an additional subfamily specific to *Caenorhabditis elegans *and outliers, which include the prokaryotic Argonautes (eubacterial and archaeal), though the latter category displays some similarity to the Piwi subfamily. Structural studies have revealed the molecular functions of Argonaute, showing that Argonaute is 'Slicer' [2,3], and that it provides anchor sites for the 5' and 3' ends of the guide RNA strand [4-10]. Thus, Argonaute is the principal protein component of RNA silencing. The structural studies also show that three-dimensional Argonaute is roughly a bi-lobal protein, with an N-terminal lobe composed of an N-domain, L1 linker region and PAZ domain (highly mobile) and a C-terminal lobe composed of MID and PIWI domains. Patel *et al*. now provide the first pictures of full length Argonaute in complex with guide and target strands [11-13], captured at multiple distinct phases of the catalytic cycle, revealing the molecular mechanisms of this slicing machine. As with other structural studies on full length Argonaute, the protein stems from a prokaryote (*Thermus thermophilus*). This is because eukaryotic Argonautes from any subfamily are difficult to obtain in the quantities required for X-ray crystallography. Previous studies showed that prokaryotic Argonautes display a preference for a DNA guide strand [9,14] (their *in vivo *function is still unknown) and, accordingly, Patel and colleagues crystallized the complexes with a DNA guide and RNA targets.

## Snapshots of the slicing cycle

**Figure 1:**
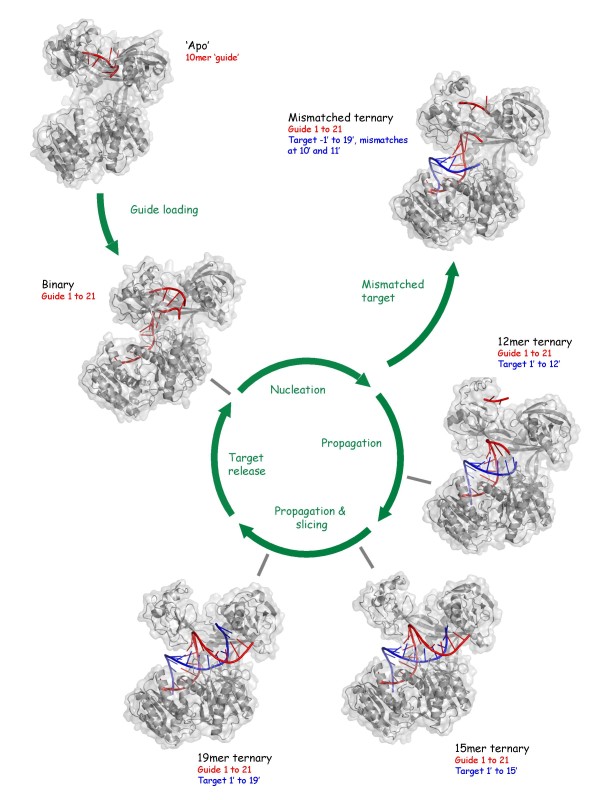
**Snapshots of the slicing cycle**. The figure shows key structures determined [11-13] and the stages of Ago-mediated silencing they most closely represent. *Thermus thermophilus *Argonaute (TtAgo) is shown in grey, guide DNA in red and target RNA in blue. The designations given for the structures (for example, 'Apo') are used in the main text and in the other figures. The positions covered by the guide or target strands are listed (numbering from the guide 5' end). Protein data bank (PDB) codes for the structures are as follows: 'Apo' - 3DLB[11], Binary - 3DLH[11], Mismatched ternary - 3F73[12], 12 mer ternary - 3HO1[13], 15 mer ternary - 3HJF[13], 19 mer ternary - 3HK2[13]. The figure, together with Figures 2 and 3, was produced using Pymol [40].

The structures and the stages in the Argonaute cycle that they most closely represent are summarized below and shown in Figure 1:

• The binary complex [11], consisting of *Thermus thermophilus *Argonaute (TtAgo) and a 21 mer DNA guide strand. The structure is a molecular picture of the substrate-free Argonaute/guide complex, primed for target recognition.

• A mismatched ternary complex [12], consisting of TtAgo, DNA guide and a 20 mer RNA target bearing mismatches to the guide at the 10' and 11' positions (numbered from the 5' end of the guide). With mismatches surrounding the scissile phosphate (between nucleotides 10' and 11'), the structure is representative of a slicing-inactive complex, reminiscent of a microRNA (miRNA)/passenger or miRNA/messenger RNA (mRNA) target complex.

• 12 mer, 15 mer and 19 mer ternary complexes [13], consisting of mutated TtAgo (to inactivate slicing), DNA guide and fully complementary RNA target strands of increasing length (12, 15 and 19 nucleotides). It is thought that the target interaction nucleates within positions 2-8 of the guide (the seed region), following which the duplex propagates towards the 3' end of the guide [14-18]. These structures, therefore, provide insight into the nucleation, propagation and slicing stages of 'active' slicer complexes.

• Supplementing these structures, Patel and colleagues also solved a binary complex with a short 10 mer DNA 'guide' [11], providing some insight into the conformation of an apo-TtAgo and, though not displayed in Figure 1, a second independent crystal form of the mismatched ternary complex [13] and unmutated 19 mer ternary complexes obtained in the presence of high concentrations of magnesium [13], elucidated to capture the active slicing geometry of the catalytic site.

## Anchoring of the guide in Argonaute

### 5' and 3' end tethering

Previous structural studies on isolated domains of Argonaute identified highly conserved anchor sites for the 5' and 3' ends of the guide strand [4-10]. The TtAgo structures reveal these to be key anchor sites in the full length protein, defining the orientation of the guide strand within Argonaute. The structures provide a second example of the geometry of the 5' binding pocket (after AfPiwi [9,10]), at the junction of the MID and PIWI domains, confirming the previously-described configuration involving a metal ion coordinated to the C-terminal carboxylate of the Argonaute polypeptide and the first (5') and third phosphates of the guide strand. Curiously, in TtAgo, an arginine replaces tyrosine in the highly conserved quartet of residues contacting the 5' phosphate (YKQK), a switch so far unique to this protein. Tethering of the 3' end in the PAZ domain, although not a feature of all the complexes (discussed below), mirrors the interactions observed previously in structures involving eukaryotic PAZ domains [4-8].

### Support for the two-state model of Ago function

**Figure 2:**
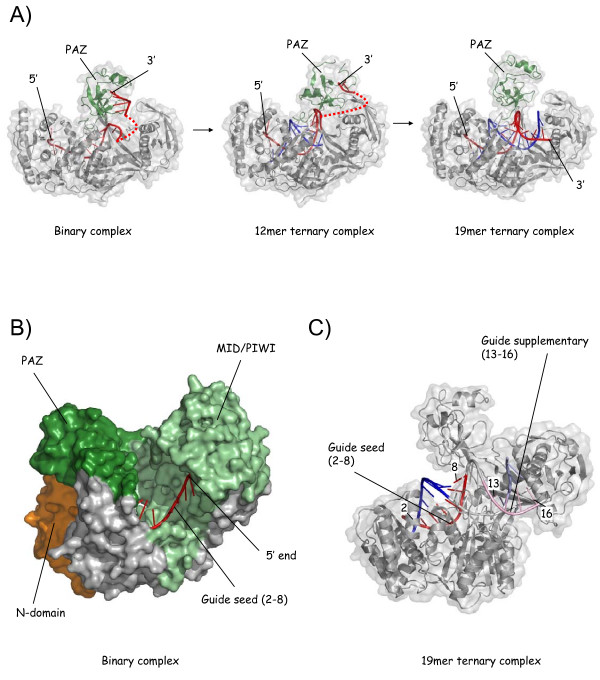
**The 'two-state' mechanism and insight into target recognition**. (A) Confirmation of the 'two-state' model for guide anchoring. The figure displays the binary complex [11] and early (12 mer ternary) and late (19 mer ternary) propagation complexes [13], illustrating release of the 3' end of the guide (red) from PAZ (green) upon formation of a sufficient number of guide - target base pairs (12-14). Dotted lines indicate connectivity where atoms are invisible in the structures (due to disorder). (B) Exposure of the seed nucleotides in the binary complex. The figure displays the binary complex [11] with the seed nucleotides (red) in a cavity in the narrowed nucleic acid binding channel in TtAgo. The backbone in a quasi-helical conformation is bedded against the MID and PIWI domains (pale green) whilst the base edges face outwards. (C) Regions of seed and 3' supplementary base pairing in the 19 mer ternary complex. The figure highlights base pairing in the 19 mer ternary complex [13] mediated by positions 2-8 of the guide (the seed, in red, with corresponding target nucleotides in blue) and positions 13-16 of the guide (positions of 3' supplementary pairing [26], with guide in pink and target in pale blue).

Despite the multiple anchoring interactions at the 5' and 3' binding sites, the structures reveal a dynamic cycle of guide end tethering. Remarkably, the structures support directly a previously proposed scheme known as the 'two-state' model [16]. In this model, the 3' end of the guide switches on and off PAZ during the catalytic cycle, being anchored, inaccessibly, in the binary complex and released in a ternary complex. (The 5' end remains fixed.) The model helps to explain the preferential association of the target with the 5' section of the guide [17,19]. It would also lead to protection of the 3' end of the guide when single-stranded in the binary complex, whilst facilitating duplex annealing during the propagation stages of guide/target duplex formation. By comparing the structure of the binary complex with the structures of the complementary 'propagation' complexes containing target strands of increasing length [13], Patel and colleagues show indeed that TtAgo fixes both ends of the guide in the binary complex, and that the 3' end of the guide is released in a ternary complex - once a requisite number of base pairs are formed (15 mer ternary complex) (Figure 2A). Presumably, the propagating duplex accumulates sufficient annealing energy to wrest the 3' end of the guide from the binding site in PAZ. It is noteworthy that this model does not apply when the target strand contains mismatches to the guide at positions 10 and 11 (mismatched ternary complex) [12]. In this ternary structure, PAZ retains the guide 3' end (Figure 1).

### Base-specificity for the 5' nucleotide of the guide

Argonaute proteins are carriers for small RNAs almost irrespective of sequence, which is reflected in the almost complete absence of base-specific contacts in any of the TtAgo complexes. The notable exception is the 5' nucleotide of the guide, which is frequently uridine in miRNAs and Piwi-interacting RNAs (piRNAs) and, strikingly, is capable of directing the sorting of small RNAs into different Argonaute family members in *Arabidopsis *(whether uridine, adenosine or cytidine) [20-22]. The high-resolution structures of the ternary propagation complexes [13] provide the first insight into how this selectivity could be mediated, through the identification of base-specific contacts between the Ago scaffold and the 5' nucleotide. Asn413 in TtAgo, whose side chain contacts directly the thymine base (DNA guide), is semi-conserved across the Argonaute family (as asparagine, glutamine or threonine). Interestingly, the *Arabidopsis *Argonautes display unusual diversity at this position, substituting either glutamine (in Ago5), cysteine (in Ago6), or leucine, alanine and valine (other Agos). A more complete picture will, however, require a eukaryotic Ago - guide RNA structure, as the prokarytic Argonautes are substantially diverged and reveal only the most conserved interactions.

## Target recognition

A substantial body of evidence indicates that the primary region within the guide for target recognition, in both small interfering RNA(siRNA) and miRNA-mediated silencing, is nucleotides 2 to 7/8 (as measured from the 5' end) [18]. This region, known as the seed sequence [23], provides the specificity in target selection, and a greater portion of the target binding energy [17,19,24]. Indeed, in some instances, complementarity over the seed region can be sufficient to mediate silencing [25]. However, within animal miRNAs, where central and 3' complementarity is not required for *slicing*, a beneficial effect for 3' pairing is still detectable [24-26], particularly between positions 13 and 16 [26], implying a role in binding stability.

What can we learn from the structures about target recognition? The binary complex structure, representing the guide and Argonaute poised to recognize a target as part of, say, RISC or RITS, is very informative [11]. Crucially, the seed nucleotides (2-8), despite being single stranded, arrange in an ordered, quasi-helical arrangement within a canyon in TtAgo, bedded against the wall of the MID/PIWI lobe (Figure 2B). The phosphodiester backbone anchors the seed to the protein, whilst the base edges of nucleotides 2 - 6 face outwards, exposed to the exterior, positioned to capture a target. (Nucleotides 7 and 8 are ordered but partially buried.) By contrast, in the 3' half of the guide, nucleotides 12 to 17 are disordered (invisible).

What are the energetic consequences of this arrangement for target recognition? Barford and colleagues have recently provided insight into the energetics of the seed-target nucleation stage of target recognition, employing a technique known as isothermal titration calorimetry (ITC) [27]. This method uses the heat absorbed or released during a binding event to provide highly accurate binding affinities and delineation of the relative contributions of enthalpy and entropy to binding. The group utilized a protein from *Archaeoglobus fulgidus *composed solely of a MID/PIWI lobe (AfPiwi), together with short RNA and DNA oligonucleotides, to recapitulate the protein/nucleic acid platform over the seed region. Notably, they observe that this platform displays a far higher affinity for a target DNA or RNA strand than is observed for a guide in isolation (an enhancement of up to ~300-fold). Thus, the tethering of the guide to the MID/PIWI lobe imbues the seed with enhanced binding properties.

One could envisage a number of mechanisms for such enhancement. The protein could make additional contacts to the target strand, supplementing those from the base pairs. Alternatively, the protein could facilitate enhancement directly through the guide strand, either via modulation of the entropy of binding, or through the enthalpic enhancement of the base pair interactions, perhaps through effects on solvent structure. Combined crystallographic and thermodynamic analysis reveals the mechanism [27]: seed-to-target binding is tighter because pre-association of the guide with the protein diminishes the entropy penalty incurred during the interaction (a disorder to order transition). The enhancement does not require any new contacts from AfPiwi to the target [27]. Thus, the pre-ordering or tethering of the guide by the protein directly establishes the enhanced binding site. The structure of the TtAgo binary complex confirms and reinforces the importance of this mechanism. As described previously, the complex displays rigid ordering of the 5' portion of the guide, whilst the 3' region is substantially disordered [11]. The asymmetry in ordering is consistent with biochemical studies showing preferential target association with the 5' portion of the guide [17,19], supplementing effects that may be derived from accessibility and duplex-compatible structure [15,16]. Thus, the structural and thermodynamic studies combine to prove a long-standing inference, that ordering of the seed by Argonaute forms the basis for favoured target recognition [14-18]. Furthermore, because Ago pre-pays some of the entropy penalty for guide/target nucleation, the mechanism links the energetics of guide loading into Ago with those of target recognition.

The structures also provide insight into target recognition fidelity. The TtAgo ternary complexes show that the guide/target duplex forms a continuous A-form-like duplex over the seed region, with numerous contacts from Ago to the phosphodiester backbone of the guide strand but, notably, no hydrogen-bonding contacts to the target strand [12,13]. This is compatible with the requirement to retain the guide strand within Ago and, in a multiple-turnover situation [19,28], release the target strand. Furthermore, this asymmetry reflects in the tolerance of TtAgo for bulges in either the guide or target over the seed region. Assays show that a bulge in the guide at position 5 abolishes slicing, whereas a bulge at a similar position in the target has little effect [12]. Presumably, the tight network of interactions from TtAgo which are restraining the guide restrict the capacity for distortion of the guide in the guide/target duplex. This may have implications for our understanding of miRNA target recognition, suggesting that target sites containing seed region bulges (for example, the 5' *let-7 *site in the *lin-41 *3' UTR in *C. elegans *[29]) could be better tolerated than those with seed region deletions (or guide bulges). The selective restraint of one strand would explain the apparent capacity of Argonaute to increase destabilization of mismatches (such as G:U wobbles) within the seed region [24,25,27].

The structures of the ternary complexes provide a starting point for the understanding of the contribution of 3' base pairs to animal microRNA target recognition stability [24-26]. The structure of the complementary 19 mer ternary complex [13] reveals, most unexpectedly, that TtAgo blocks base pairing of a complementary guide-target duplex after position 16 (Figure 2C; discussed further later). Duplex forms only between positions 2 and 16. This may, in part, explain why position 16 describes the 3' limit for significant 3' pairing in miRNA target recognition [26]. The mismatched ternary structure, *prima facie *more relevant to animal miRNA/target interactions, shows an alternative and distinct conformation for the 3' portion of the guide/target duplex [12]. Here, following mismatches at positions 10 and 11, the guide/target duplex disorders between positions 12 and 19, with PAZ retaining the 3' end of the guide. The significance of 3' pairing in this structure, if any, is more difficult to ascertain. It is likely that the two structures (complementary and mismatched) provide examples of two possible conformations of the 3' portion of the guide/target duplex within Ago, adopted according to the individual circumstances of a particular recognition event.

Outside of a particular guide strand footprint, one of the major contributors to animal miRNA target site efficacy is proximity to a second site [26,30], with seed spacings of as little as eight nucleotides resulting in the synergistic enhancement of silencing [26]. One explanation for this effect is cooperative interactions between silencing complexes, but the TtAgo crystal structures provide no obvious basis for this. However, the 3' disordering of the target observed in the mismatched ternary structure [12], despite the presence of complementary base pairs, in principle frees up the target and provides an opportunity for a second silencing complex to interface via a seed sequence at close proximity.

## The Slicer catalytic site

**Figure 3:**
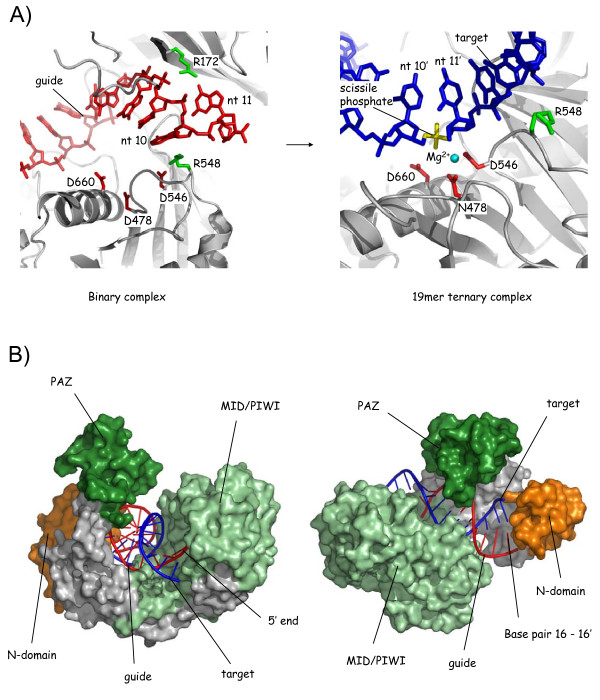
**The slicer catalytic site and a role for the N-domain as a duplex wedge**. (A) Assembly of the slicer site. The figure show zoomed-in views of the slicer catalytic site, in the binary [11] and 19 mer ternary [13] complexes. The figure illustrates conformational changes that accompany complementary target strand annealing. Key residues are highlighted. The guide is omitted in the right panel for clarity. The 19 mer ternary complex structure shown was obtained using an N478 catalytic site mutant [13] and, therefore, the structure is partially-distorted with only a single catalytic magnesium ion. (B) The N-domain as a duplex wedge. Two views of the 19 mer ternary complex [13] illustrating encapsulation by TtAgo of the fully annealed complementary guide/target duplex and blockage at position 16 by the N-domain (orange). The view on the left shows the duplex from the guide 5' end and the widened nucleic acid binding channel between the MID/PIWI lobe (pale green) and PAZ (dark green). The view on the right shows the same structure rotated and from above, illustrating enclosure of the duplex and the abrupt arrest at position 16 (guide)/16' (target). Nucleotides 17-21 of the guide and 17'-19' of the target are invisible, presumably disordered, though it is assumed they bypass either side of the N-domain.

Comparison of the slicer catalytic site in TtAgo between the binary and ternary complexes reveals a highly distorted catalytically incompetent site in the binary complex, transitioning to a catalytically competent form upon annealing of a complementary substrate (12 mer, 15 mer and 19 mer ternary complexes) (Figure 3A). An interesting and unexpected feature is the involvement of two arginine residues (R172 and R548) which in the binary complex appear to stabilize a disruption in the quasi-helical nucleotide stack of the guide between the 10th and 11th nucleotides (that is, at the cleavage locus), resulting in their orthogonal arrangement. In the ternary complexes, by contrast, R548 is displaced, allowing the continuation of an unhindered duplex. However, these residues are almost entirely unconserved at these positions in the Argonaute protein family (apart from in some other prokaryotes), which is puzzling for residues that appear clearly to be mechanistically significant in TtAgo.

Mismatches and mutated catalytic residues, used to capture unsliced ternary complexes, distort the catalytic geometry of the active site and so, in a notable feat of crystallography, Patel and colleagues also obtained diffracting crystals of wild-type TtAgo in the presence of a guide and fully-complementary target [13]. The structures present the catalytic geometry of the slicer site for the first time. The slicer residues (DDD), catalytic magnesium ions (x2) and target RNA strand superimpose closely with the structure of an RNase H catalytic complex, from *Bacillus halodurans *[13,31]. Thus, as predicted originally from the fold of the PIWI domain [2,3], slicer employs RNase H-like chemistry to execute slicing of the target, or passenger, RNA strand.

## PAZ as an inhibitor of slicing?

Slicer assays using 3'-truncated target strands (3' relative to the *guide*), which thereby test the slicing capacity of ternary propagation-equivalent complexes, suggest an interesting and novel mode of slicing regulation within Ago [13]. The assays show that truncation too far in the 3' direction inhibits slicing (at or 5' to position 15), implying that slicing is inhibited during the earlier stages of propagation. This is puzzling when these complexes contain a fully complementary duplex covering the seed and central regions. On the other hand, 3'-truncation of the *guide *down to position 9 does not significantly affect slicing - despite the absence of rigid duplex geometry around the scissile phosphate [12]. The switch in activity in the propagation complexes correlates approximately with a change in conformation observed in the ternary complex structures (12 mer and 15 mer), characterized by the release of the 3' end of the guide from PAZ (Figure 2A). Patel and colleagues suggest, therefore, that perhaps PAZ must release the 3' end of the guide in order for slicing to be allowed [13]. This would represent a switch from an inactive to an active slicing conformation. Tethering of the 3' end of the guide could influence slicing through the conformation of Ago and/or the guide/target duplex. Notably for the mechanism of slicing inhibition in animal microRNA complexes, PAZ retains the 3' end of the guide in the mismatched ternary complex. Significantly, this could constitute a key aspect of the mechanism of slicing inhibition in animal microRNA effector complexes.

## The N-domain as a duplex wedge?

The 19 mer ternary structure, representing the most complete propagation complex (positions 1 - 19), reveals formation of a regular A-form guide/target duplex but shows, unexpectedly, that the helix terminates at position 16, blocked head-on by the N-domain of TtAgo [13] (Figure 3B). The remaining nucleotides are invisible but the strands are presumed to be separated, passing on either side of the N-domain. As such, the N-domain functions as a wedge, interceding in the trajectory of the annealed guide/target duplex. This could facilitate recycling after slicing, restricting annealing to six base pairs 3' of the scissile phosphate, a mechanism that would be particularly important with longer guide strands (for example, with Piwi-interacting RNAs [piRNAs]). Thus, TtAgo pre-unwinds both ends of the annealed guide/target duplex: position 1 at the 5' end of the guide and positions 17-21 at the 3' end. An α-helix in the N-domain mediates duplex termination, contributing two (unconserved) residues (Y43 and P44) to stack on the end of the duplex; the structure is reminiscent of that which caps and divides the 5' end.

## Multiple turnover activity

Eukaryotic RISC and recombinant eukaryotic Argonaute display distinct bi-phasic cleavage kinetics under multiple turnover conditions (excess target strand), with an initial relatively rapid burst of activity followed by a slower 'steady-state' phase [17,19,32-34]. This is particularly notable for recombinant eukaryotic Argonaute (human Ago2), which displays very slow steady-state kinetics [33]. The rapid burst of activity probably corresponds to the first single-turnover stage; extrapolation of the steady state rate curve to the ordinate axis yields approximately the concentration of the enzyme in the reaction [17,19,32-34]. This suggests that, under multiple turnover conditions, the recycling stage is the rate-limiting step. Mismatches [19] or competitive blocking [17] at the 3' end of the guide can alleviate this effect, indicating that product release (rather than, say, a regeneration step after product release) is the limiting stage. This may be expected when RNA strand-strand interactions are very tight, with theoretical affinities for ~10 mer duplexes in the nM range (dissociation constants). Under some circumstances, adenosine triphosphate (ATP) can also alleviate the effect [19] suggesting that, *in vivo*, an ATP-assisted enzyme (such as a helicase, or Hsp90 [35-37]) will contribute to recycling. However, at the same time it is clear that RISC as a complex does, overall, substantially weaken the guide/target interaction, because estimated affinities for guide binding to RISC indicate a massively weaker interaction than for the equivalent guide/target interaction in isolation [17,19].

Patel and colleagues show that *Thermus thermophilus *Argonaute is, in isolation, a multiple turnover enzyme [13]. In contrast to recombinant hAgo2 [33], the enzyme does not appear to display a recycling rate-limiting step, with no evidence for bi-phasic kinetics under multiple turnover conditions (excluding a lag phase) or a substantial difference in rate between single- and multiple-turnover conditions. Does this mean that the structural interactions and rearrangements observed upon formation of the slicing-competent complexes reveal the basis for facilitated product release? Unfortunately, this is not so obviously the case, because the TtAgo cleavage assays are conducted at high temperature (75°C, as the protein stems from a thermophile) which significantly weakens nucleic acid strand interactions, and multiple turnover is conducted using DNA guide and target strands, which in addition interact more weakly than their RNA counterparts. In fact, the theoretical affinities of the sliced DNA cleavage products for the guide, at this temperature, lie between 100 and 400 mM (dissociation constants) (10^5^-fold higher than the concentration of nucleic acid in the reactions). Nonetheless, the TtAgo structures provide some clues as to the mechanisms adopted by eukaryotic Argonautes to manage the requirement to function as catalytic enzymes and cleave multiple substrates. First, it is quite striking that there are almost no direct hydrogen-bonding contacts from TtAgo to the target strand across the whole length of the substrate (apart from around the scissile phosphate, which are likely to function to fine-tune positioning of the target with respect to the catalytic residues in the Ago scaffold.) Second, as already discussed, both ends of the formed guide/target duplex are splayed by Ago, which provides a starting point for unwinding. Indeed, one of these free single-stranded ends may be the initial substrate for an ATP-assisted helicase to mediate unwinding. Finally, the extraordinary rearrangements in the Ago scaffold coupled to duplex propagation, evidenced by the pivoting of the Ago domains around an uninterrupted, undistorted guide/target duplex, suggest tensions accumulated and overcome during propagation which may be exploited to eject the sliced target strand, once the duplex is compromised by the central slicing event.

## Conclusions and prospects

The structures of *T. thermophilus *Argonaute in complex with guide and target strands presented by Patel and colleagues provide molecular insight into the central engine of RNA silencing. The structures confirm previously hypothesized mechanisms, including the 'two-state' model for guide tethering, and reveal new ones, such as a potential role for PAZ as an internal inhibitor of slicing, and a role for the N-domain as duplex wedge at the 3' end of the guide. In addition, the structures provide new molecular detail in, for instance, 5' nucleotide recognition of the guide, target recognition via the seed sequence and the chemistry of the slicing reaction. These insights are relevant to siRNA, miRNA and piRNA mediated silencing, suggest opportunities for the mutagenesis of eukaryotic Argonautes and provide a molecular basis for the enhancement via chemical modification of reagent and therapeutic siRNAs. Coupled to thermodynamic and kinetic studies from other groups, the structures now provided a detailed mechanistic understanding of the operation of Argonaute. However, a great deal remains to be achieved. These challenges can be viewed in two broad areas. First is the requirement to understand, via structures, the molecular details of *eukaryotic *Argonautes, from all sub-families. Second is the requirement to understand how other proteins interface with Argonaute, including in RISC, RITS and the Sago and piRNA effector complexes. Very recently, the first progress in this area has been reported by Doudna, Nogales, Wang and colleagues [38], and Macrae and colleagues [39], who describe the first electron microscopy reconstructions of human Dicer and the RISC-loading complex. Nonetheless, the prokaryotic Argonautes provide a foundation for this work, whose amenability to high resolution X-ray crystallography have revealed the dynamicity of Ago at the heart of the slicing catalytic cycle.

## Abbreviations

AfPiwi: *Archaeoglobus fulgidus *Piwi; Ago: Argonaute; ATP: adenosine triphosphate; ITC: isothermal titration calorimetry; mRNA: messenger RNA; miRNA: microRNA; piRNA: piwi-interacting RNA; RISC: RNA-induced silencing complex; RITS [complex]: RNA-induced initiation of transcriptional gene silencing [complex]; siRNA: small interfering RNA; TtAgo: *Thermus thermophilus *Argonaute.

## Competing interests

The author declares that they have no competing interests.

## References

[B1] Carmell MA Xuan Z Zhang MQ Hannon GJ The Argonaute family: tentacles that reach into RNAi, developmental control, stem cell maintenance, and tumorigenesis Genes Dev 2002 16 21 2733 2742 10.1101/gad.1026102 12414724

[B2] Song JJ Smith SK Hannon GJ Joshua-Tor L Crystal structure of Argonaute and its implications for RISC slicer activity Science 2004 305 5689 1434 1437 10.1126/science.1102514 15284453

[B3] Parker JS Roe SM Barford D Crystal structure of a PIWI protein suggests mechanisms for siRNA recognition and slicer activity Embo J 2004 23 24 4727 4737 10.1038/sj.emboj.7600488 15565169 PMC535097

[B4] Lingel A Simon B Izaurralde E Sattler M Structure and nucleic-acid binding of the *Drosophila *Argonaute 2 PAZ domain Nature 2003 426 6965 465 469 10.1038/nature02123 14615801

[B5] Yan KS Yan S Farooq A Han A Zeng L Zhou MM Structure and conserved RNA binding of the PAZ domain Nature 2003 426 6965 468 474 10.1038/nature02129 14615802

[B6] Song JJ Liu J Tolia NH Schneiderman J Smith SK Martienssen RA Hannon GJ Joshua-Tor L The crystal structure of the Argonaute2 PAZ domain reveals an RNA binding motif in RNAi effector complexes Nat Struct Biol 2003 10 12 1026 1032 10.1038/nsb1016 14625589

[B7] Ma JB Ye K Patel DJ Structural basis for overhang-specific small interfering RNA recognition by the PAZ domain Nature 2004 429 6989 318 322 10.1038/nature02519 15152257 PMC4700412

[B8] Lingel A Simon B Izaurralde E Sattler M Nucleic acid 3'-end recognition by the Argonaute2 PAZ domain Nat Struct Mol Biol 2004 11 6 576 577 10.1038/nsmb777 15156196

[B9] Ma JB Yuan YR Meister G Pei Y Tuschl T Patel DJ Structural basis for 5'-end-specific recognition of guide RNA by the *A. fulgidus *Piwi protein Nature 2005 434 7033 666 670 10.1038/nature03514 15800629 PMC4694588

[B10] Parker JS Roe SM Barford D Structural insights into mRNA recognition from a PIWI domain-siRNA guide complex Nature 2005 434 7033 663 666 10.1038/nature03462 15800628 PMC2938470

[B11] Wang Y Sheng G Juranek S Tuschl T Patel DJ Structure of the guide-strand-containing Argonaute silencing complex Nature 2008 456 7219 209 213 10.1038/nature07315 18754009 PMC4689319

[B12] Wang Y Juranek S Li H Sheng G Tuschl T Patel DJ Structure of an argonaute silencing complex with a seed-containing guide DNA and target RNA duplex Nature 2008 456 7224 921 926 10.1038/nature07666 19092929 PMC2765400

[B13] Wang Y Juranek S Li H Sheng G Wardle GS Tuschl T Patel DJ Nucleation, propagation and cleavage of target RNAs in Ago silencing complexes Nature 2009 461 754 761 10.1038/nature08434 19812667 PMC2880917

[B14] Yuan YR Pei Y Ma JB Kuryavyi V Zhadina M Meister G Chen HY Dauter Z Tuschl T Patel DJ Crystal structure of *A. aeolicus *Argonaute, a site-specific DNA-guided endoribonuclease, provides insights into RISC-mediated mRNA cleavage Mol Cell 2005 19 3 405 419 10.1016/j.molcel.2005.07.011 16061186 PMC4689305

[B15] Filipowicz W RNAi: the nuts and bolts of the RISC machine Cell 2005 122 1 17 20 10.1016/j.cell.2005.06.023 16009129

[B16] Tomari Y Zamore PD Perspective: machines for RNAi Genes Dev 2005 19 5 517 529 10.1101/gad.1284105 15741316

[B17] Ameres SL Martinez J Schroeder R Molecular basis for target RNA recognition and cleavage by human RISC Cell 2007 130 1 101 112 10.1016/j.cell.2007.04.037 17632058

[B18] Bartel DP MicroRNAs: target recognition and regulatory functions Cell 2009 136 2 215 233 10.1016/j.cell.2009.01.002 19167326 PMC3794896

[B19] Haley B Zamore PD Kinetic analysis of the RNAi enzyme complex Nat Struct Mol Biol 2004 11 7 599 606 10.1038/nsmb780 15170178

[B20] Mi S Cai T Hu Y Chen Y Hodges E Ni F Wu L Li S Zhou H Long C Sorting of small RNAs into *Arabidopsis *Argonaute complexes is directed by the 5' terminal nucleotide Cell 2008 133 1 116 127 10.1016/j.cell.2008.02.034 18342361 PMC2981139

[B21] Montgomery TA Howell MD Cuperus JT Li D Hansen JE Alexander AL Chapman EJ Fahlgren N Allen E Carrington JC Specificity of ARGONAUTE7-miR390 interaction and dual functionality in TAS3 trans-acting siRNA formation Cell 2008 133 1 128 141 10.1016/j.cell.2008.02.033 18342362

[B22] Takeda A Iwasaki S Watanabe T Utsumi M Watanabe Y The mechanism selecting the guide strand from small RNA duplexes is different among Argonaute proteins Plant Cell Physiol 2008 49 4 493 500 10.1093/pcp/pcn043 18344228

[B23] Lewis BP Shih IH Jones-Rhoades MW Bartel DP Burge CB Prediction of mammalian microRNA targets Cell 2003 115 7 787 798 10.1016/S0092-8674(03)01018-3 14697198

[B24] Doench JG Sharp PA Specificity of microRNA target selection in translational repression Genes Dev 2004 18 5 504 511 10.1101/gad.1184404 15014042 PMC374233

[B25] Brennecke J Stark A Russell RB Cohen SM Principles of microRNA-target recognition PLoS Biol 2005 3 3 e85 10.1371/journal.pbio.0030085 15723116 PMC1043860

[B26] Grimson A Farh KK Johnston WK Garrett-Engele P Lim LP Bartel DP MicroRNA targeting specificity in mammals: determinants beyond seed pairing Mol Cell 2007 27 1 91 105 10.1016/j.molcel.2007.06.017 17612493 PMC3800283

[B27] Parker JS Parizotto EA Wang M Roe SM Barford D Enhancement of the seed-target recognition step in RNA silencing by a PIWI/MID domain protein Mol Cell 2009 33 2 204 214 10.1016/j.molcel.2008.12.012 19187762 PMC2642989

[B28] Hutvagner G Zamore PD A microRNA in a multiple-turnover RNAi enzyme complex Science 2002 297 5589 2056 2060 10.1126/science.1073827 12154197

[B29] Vella MC Choi EY Lin SY Reinert K Slack FJ The *C. elegans *microRNA let-7 binds to imperfect let-7 complementary sites from the lin-41 3'UTR Genes Dev 2004 18 2 132 137 10.1101/gad.1165404 14729570 PMC324419

[B30] Saetrom P Heale BS Snove O Jr Aagaard L Alluin J Rossi JJ Distance constraints between microRNA target sites dictate efficacy and cooperativity Nucleic Acids Res 2007 35 7 2333 2342 10.1093/nar/gkm133 17389647 PMC1874663

[B31] Nowotny M Gaidamakov SA Crouch RJ Yang W Crystal structures of RNase H bound to an RNA/DNA hybrid: substrate specificity and metal-dependent catalysis Cell 2005 121 7 1005 1016 10.1016/j.cell.2005.04.024 15989951

[B32] Martinez J Tuschl T RISC is a 5' phosphomonoester-producing RNA endonuclease Genes Dev 2004 18 9 975 980 10.1101/gad.1187904 15105377 PMC406288

[B33] Rivas FV Tolia NH Song JJ Aragon JP Liu J Hannon GJ Joshua-Tor L Purified Argonaute2 and an siRNA form recombinant human RISC Nat Struct Mol Biol 2005 12 4 340 349 10.1038/nsmb918 15800637

[B34] Forstemann K Horwich MD Wee L Tomari Y Zamore PD Drosophila microRNAs are sorted into functionally distinct Argonaute complexes after production by dicer-1 Cell 2007 130 2 287 297 10.1016/j.cell.2007.05.056 17662943 PMC2686109

[B35] Liu J Carmell MA Rivas FV Marsden CG Thomson JM Song JJ Hammond SM Joshua-Tor L Hannon GJ Argonaute2 is the catalytic engine of mammalian RNAi Science 2004 305 5689 1437 1441 10.1126/science.1102513 15284456

[B36] Tahbaz N Kolb FA Zhang H Jaronczyk K Filipowicz W Hobman TC Characterization of the interactions between mammalian PAZ PIWI domain proteins and Dicer EMBO Rep 2004 5 2 189 194 10.1038/sj.embor.7400070 14749716 PMC1298981

[B37] Pare JM Tahbaz N Lopez-Orozco J LaPointe P Lasko P Hobman TC Hsp90 regulates the function of Argonaute 2 and its recruitment to stress granules and P-bodies Mol Biol Cell 2009 20 14 3273 3284 10.1091/mbc.E09-01-0082 19458189 PMC2710822

[B38] Wang HW Noland C Siridechadilok B Taylor DW Ma E Felderer K Doudna JA Nogales E Structural insights into RNA processing by the human RISC-loading complex Nat Struct Mol Biol 2009 16 11 1148 1153 10.1038/nsmb.1673 19820710 PMC2845538

[B39] Lau PW Potter CS Carragher B Macrae IJ Structure of the human Dicer-TRBP complex by electron microscopy Structure 2009 17 10 1326 1332 10.1016/j.str.2009.08.013 19836333 PMC2880462

[B40] Pymol home page http://www.pymol.org/

